# Supplementary material for: Parasitic Effects on the Congenital Transmission of Trypanosoma cruzi in Mother–Newborn Pairs
Source: Microorganisms. 2024 Jun 20;12(6):1243. doi: 10.3390/microorganisms12061243 (PMC11206037; doi:10.3390/microorganisms12061243)
Supplement: Supplementary file 1 [file microorganisms-12-01243-s001.zip › microorganisms-3049359-supplementary.pdf]

UNIVERSIDAD MAYOR DE SAN ANDRÉS

COMITÉ DE ÉTICA DE LA INVESTIGACIÓN DE LA UMSA

CEI - UMSA

CERTIFICADO DE AVAL ÉTICO

A quien corresponda:

El comité de Ética de la Investigación de la Universidad Mayor de San Andrés (CEI-UMSA) tiene a bien informar que fue presentado para su evaluación y aval ético, el Proyecto titulado: **"Enfermedad de Chagas congénita"**, financiado por el "Institut de Recherche pour le développement" (IRD), en el marco de "Jeunes Equipes Associées a l`IRD, por el laboratorio de Inmunoparasitología, Facultad de Medicina, Enfermería, Nutrición y Tecnología Médica, cuya investigadora principal es la Dra. Celeste Rodriguez.

Dicho proyecto fue evaluado bajo la normativa internacional en ética de la investigación (Pautas CIOMS/OMS, Helsinki/AMM) en la que se incluyen los criterios éticos que se deben tomar en cuenta para investigaciones que involucran seres humanos:

1. Validez científica (proyecto que cumpla con todo el rigor de la metodología científica)
2. Selección equitativa de la muestra (tipo de individuos que entran al estudio, tomando en cuenta principalmente, a grupos vulnerables)
3. Validez social (pertinencia, atingencia y relevancia del proyecto)
4. Relación Riesgo/Beneficio) donde el riesgo sea mínimo y mayor el beneficio para los sujetos del estudio)
5. La hoja de Información y el Consentimiento Informado (documentos redactados de una manera clara, comprensible y lo suficiente informativos para el participante, que reflejen el respeto a la autonomía)

Una vez verificadas las correcciones hechas por el equipo investigador, en base a las observaciones del CEI-UMSA, es que se certifica que el mencionado proyecto cumple con todos los requisitos éticos arriba mencionados, por lo que se decide otorgar el **CERTIFICADO DE AVAL ETICO** al proyecto **"Enfermedad de Chagas congénita"**, el mismo puede proseguir con la evaluación determinada.

Dra. Mercedes **VILLENA CABRERA**
COORDINADORA a.i. CEI-UMSA

Ing. Carlos **ESPAÑA V.**
PRESIDENTE CEI-UMSA

La Paz, 14 de Septiembre de 2008

c.c. Interesado
Arch. CEI-UMSA

Av. Villazón N° 195, Monoblock, Piso 1 Telf. (591-2) 2440493
e-mail: cei@umsa.bo – La Paz - Bolivia
